# Development and validation of a nomogram to predict the risk of deep venous thrombosis after hip replacement in elderly patients with femoral neck fracture

**DOI:** 10.3389/fsurg.2026.1816700

**Published:** 2026-07-30

**Authors:** Fanghui Zhang, Jibin Chen, Te Li, Yiquan Cheng, Zhi Li

**Affiliations:** 1 Department of Orthopedics, The Sixth Hospital of Wuhan, Affiliated Hospital of Jianghan University, Wuhan, China; 2 Department of Orthopedics, General Hospital of Chinese PLA Central Theater Command, Wuhan, China; 3 The First School of Clinical Medicine, Southern Medical University, Guangzhou, China; 4 School of Medicine, Wuhan University of Science and Technology, Wuhan, China

**Keywords:** deep vein thrombosis, femoral neck fracture, hip replacement, prediction model, risk factors

## Abstract

**Background:**

The rising global aging population has led to more femoral neck fractures (FNF) in the elderly, posing a significant public health challenge. FNF patients undergoing hip replacement face a higher risk of deep vein thrombosis (DVT), which, if untreated, can lead to severe complications like pulmonary embolism. Without preventive measures, the incidence of DVT after major surgery is notably high.

**Methods:**

A total of 906 elderly FNF patients who underwent hip arthroplasty between January 2013 and December 2023 were enrolled from the General Hospital of Chinese PLA Central Theater Command. Patients were randomly allocated to a training set (*n* = 635, 70%) and a validation set (*n* = 271, 30%). The occurrence of DVT in the training set was designated as the outcome variable. Potential predictors were initially screened via univariate analysis and Least Absolute Shrinkage and Selection Operator (LASSO) regression. Subsequently, significant independent variables were incorporated into a multivariate logistic regression model to identify predictors and construct a nomogram prediction model. This model underwent internal validation using the validation set. Model performance was evaluated using the area under the receiver operating characteristic (ROC) curve (AUC), calibration curve, Hosmer–Lemeshow goodness-of-fit test, and decision curve analysis (DCA).

**Ethics:**

This retrospective study was approved by the Ethics Committee of the General Hospital of Central Theater Command of PLA ([2024] 074-01) and was conducted in accordance with the Declaration of Helsinki and its later amendments. All patient data were retrospectively collected from hospital medical records and anonymized before analysis.

## 1 Introduction

With the global aging population, the incidence of femoral neck fracture (FNF)—a common osteoporotic fracture—has increased significantly among elderly individuals, posing a major public health challenge (1). FNF, a prevalent form of osteoporotic fracture, constitutes approximately 9% to 15% of all body fractures and 48%–54% of hip fractures in China, primarily affecting middle-aged and elderly populations (2). For older patients, hip replacement surgery can substantially enhance clinical outcomes (3). Nonetheless, recent research has highlighted an elevated risk of deep vein thrombosis (DVT) in these patients. Contributing factors include prolonged bed rest, reduced mobility, diminished metabolic function, and slower blood flow (4). DVT is characterized by the formation of solid or semi-solid clots in the deep veins and is a common and serious complication in patients with FNF, often manifesting as swelling and pain in the affected limb. If a clot becomes dislodged, it can result in pulmonary embolism (PE), a potentially life-threatening condition (5). Research has demonstrated that, in the absence of preventive interventions, the incidence of DVT following major surgical procedures ranges from 15% to 30%, while the incidence of fatal PE is between 0.2% and 0.9%. These complications pose a substantial threat to patient health and underscore the necessity for effective preventive strategies (6).

To reduce the risk of DVT, numerous studies have identified a variety of independent risk factors, including gender, age, smoking history, hyperglycemia, duration of hospital stay, surgical technique, type of anesthesia, intraoperative blood loss, body mass index (BMI), preoperative D-dimer levels, hemoglobin levels, platelet count, prothrombin time, partial thromboplastin time, and postoperative activity limitations (7–10). The relative significance and interactions of these factors remain ambiguous and necessitate further investigation. Additionally, existing DVT risk prediction models often exhibit limited clinical utility due to small sample sizes and inadequate consideration of the combined effects of these factors. Consequently, the development of a comprehensive model capable of accurately predicting the risk of DVT in patients with hip fractures following surgery is of paramount importance. Thus, developing a comprehensive and accurate predictive tool is critical for guiding personalized preventive strategies.

The least absolute shrinkage and selection operator (LASSO) regression is a potent technique for variable selection and regularization, particularly advantageous in binary classification problems. By utilizing LASSO regression's capacity to manage multicollinearity in high-dimensional data in conjunction with the classification capabilities of logistic regression, we can generate clear and interpretable predictions (11). Nomograms, as tools for visualizing predictive outcomes, significantly augment clinical applications and facilitate individualized assessments of disease risk (12). This study aimed to develop and validate an innovative DVT risk prediction model by integrating LASSO regression with binary logistic regression analysis. The model was designed to equip clinicians with a practical instrument for managing and mitigating DVT risk in patients who have undergone hip fracture surgery.

## 2 Methods

### 2.1 Patient data

We performed a retrospective analysis of patients diagnosed with FNF who underwent hip replacement surgery at the General Hospital of Chinese PLA Central Theater Command, specifically at the Wuchang and Hankou facilities, between January 2013 and December 2023. The inclusion criteria for this study were as follows: (1) FNF confirmed via x-ray or CT scan, (2) patients who received either hemiarthroplasty (HA) or total hip arthroplasty (THA), (3) age ≥60 years, and (4) absence of any prior history of vascular surgery or lower extremity vascular embolism. The exclusion criteria were as follows: (1) age <60 years, (2) presence of DVT detected preoperatively, (3) preoperative administration of anticoagulant medications, (4) presence of bleeding disorders or active bleeding, (5) diagnosis of malignant tumors or impaired liver and kidney function, (6) multiple injuries, (7) absence of critical auxiliary test results (i.e., missing any of the following preoperative examinations: lower extremity venous ultrasonography, D-dimer, hip radiograph, routine blood tests, coagulation function tests (PT, APTT), liver and renal function tests with electrolytes, and electrocardiogram or echocardiography), and (8) intraoperative mortality or mortality unrelated to DVT following surgery.

In this study, we conducted a comprehensive screening of blood vessels in the lower extremities, specifically targeting the bilateral femoral, superficial femoral, deep femoral, common femoral, anterior tibial, posterior tibial, and peroneal veins. Superficial veins, such as the great and small saphenous veins, as well as intestinal veins, were excluded due to their comparatively lower clinical significance. Experienced sonographers employed color Doppler ultrasound to assess these vessels for the presence of DVT. The clinical manifestations and ultrasound criteria for diagnosing DVT included sudden swelling and pain in the affected limb, pitting edema upon palpation, and tenderness in the femoral triangle and iliac fossa. Ultrasound indicators of DVT comprised abnormal compression of the venous lumen, low or absent echoes within the lumen, and diminished or absent blood flow signals in the affected vein. Venography was utilized to confirm DVT in patients with a high clinical suspicion but negative or inconclusive ultrasound findings, or when further confirmation was required before invasive intervention, revealing a lack of venous filling, segmental or total deep venous stenosis, roughened venous walls, irregular lumen narrowing, and the development of collateral circulation (13, 14). Informed consent was obtained from all participants, and the study was approved by the hospital's medical ethics committee. Demographic, surgical, laboratory, and clinical data were extracted from hospital medical records by two specialist physicians in conjunction with a team of trained medical professionals.

### 2.2 Statistical analysis

Statistical analyses of the collected data were conducted using SPSS software version 26.0. For data exhibiting a normal distribution, results were expressed as mea*n* ± standard deviation (*x* ± *s*), and intergroup comparisons were performed using the independent samples *t*-test. For data not conforming to a normal distribution, results were presented as median (interquartile range) [*M* (IQR)], with comparisons made via the rank sum test. Categorical variables were reported as frequencies (percentages), and differences between groups were assessed using the Chi-square test. Statistical significance was determined with a two-tailed *P*-value threshold of less than 0.05. A cohort of 906 eligible patients was randomly allocated into a training set (*n* = 635) and a validation set (*n* = 271) in a 7:3 ratio utilizing R Studio (version 4.3.2). Within the training set, DVT served as the outcome variable, and potential predictors were identified through univariate analysis and LASSO regression (15). For the LASSO regression, all continuous variables were standardized to a mean of 0 and standard deviation of 1. A 10-fold cross-validation was applied to select the penalty parameter *λ*. The optimal *λ* was chosen as the value that lies one standard error above the minimum cross-validated error, which provides a balance between fit and parsimony. Significant independent variables were subsequently incorporated into a multivariate logistic regression analysis using a forward stepwise selection method based on the likelihood ratio test (Forward LR) to identify predictors and develop a nomogram prediction model. This model underwent internal validation using the validation dataset. To assess the model's performance, Receiver Operating Characteristic (ROC) curves were plotted for both the training and validation datasets, and the Area Under the Curve (AUC) was calculated for each risk factor to evaluate the model's discriminative capacity (16). The Hosmer–Lemeshow goodness-of-fit test was employed to examine the model's fit, and a calibration curve was constructed. Furthermore, decision curve analysis (DCA) was conducted to appraise the clinical utility of the prediction model (17).

## 3 Results

### 3.1 Patient cohorts and clinicopathologic features

**Figure 1:**
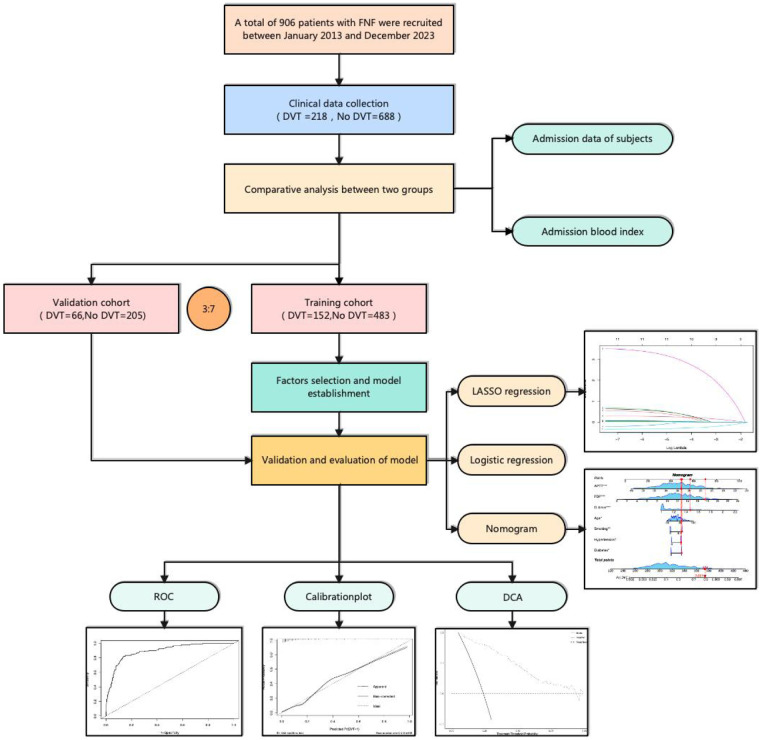
Collection procedure of all elderly patients with FNF in this study. The diagram outlines the process of patient enrollment, including inclusion and exclusion criteria, data splitting into training and validation cohorts, and the statistical analysis framework.

A comprehensive flow chart illustrating the study process is provided in Figure 1. This investigation encompassed 906 patients diagnosed with FNF from January 2013 to December 2023. A total of 906 patients were included in this study. The age ranged from 60 to 99 years, with a mean age of 74.06 ± 6.96 years and a median age of 73 years (interquartile range: 69–79). The age distribution was as follows: 60–69 years: 261 cases (28.81%); 70–79 years: 446 cases (49.23%); and ≥80 years: 199 cases (21.96%). The general characteristics of the patient cohort were statistically analyzed, and a univariate analysis was performed. Of these patients, 218 developed DVT postoperatively, corresponding to an incidence rate of 24.06%. Categorical variables were systematically classified and assigned, as shown in Table 1. Significant differences were identified between the non-DVT and DVT groups concerning age, history of diabetes and hypertension, smoking history, WBC, D-dimer levels, FIB, FDP, ESR, Caprini score, and APTT (*P* < 0.05). No significant differences were observed for other variables (*P* > 0.05), as detailed in Table 2.

**Table 1:** Valuation for categorical variables.

Variables	Assignment
Gender	0 = Female, 1 = male
Affected side	0 = Left side, 1 = right side
Damage mechanism	0 = Low energy, 1 = high energy
Diabetes	0 = No, 1 = Yes
Hypertension	0 = No, 1 = Yes
Smoking history	0 = No, 1 = Yes
Drinking history	0 = No, 1 = Yes
Coronary disease	0 = No, 1 = Yes
Operative approach	0 = HA, 1 = THA
Anesthesia type	0 = CSEA, 1 = General anesthesia
Transfusion	0 = No, 1 = Yes

**Table 2:** Patient demographics and clinicopathological characteristics.

Variables	DVT (*n* = 218)	Non-DVT (*n* = 688)	*x*^2^/*t*/*z*	*P*
Age (years)	76 (70.75, 80)	73 (69, 78)	−3.280	0.001
Gender *n* (%)			0.355	0.551
Female	75	252		
Male	143	436		
Affected side *n* (%)			0.377	0.539
Left side	100	332		
Right side	118	356		
Damage mechanism *n* (%)			0.144	0.704
High energy	108	351		
Low energy	110	337		
BMI (kg/m^2^)	26.23 ± 2.01	24.32 ± 2.17	2.275	0.132
Diabetes *n* (%)			19.436	<0.001
Yes	140	324		
No	78	364		
Hypertension *n* (%)			11.714	0.001
Yes	137	341		
No	81	347		
Smoking history *n* (%)			14.559	<0.001
Yes	140	340		
No	78	348		
Drinking history *n* (%)			1.956	0.162
Yes	124	354		
No	94	334		
Coronary disease *n* (%)			0.318	0.573
Yes	69	318		
No	122	370		
Operative approach *n* (%)			0.981	0.322
THA	112	327		
HA	106	361		
Anesthesia type *n* (%)			0.756	0.384
CSEA	95	223		
General anesthesia	123	365		
Transfusion *n* (%)			1.666	0.197
Yes	112	319		
No	106	369		
Injury-surgery time (days)	5 (4, 6)	5 (3, 6)	−0.457	0.647
ALB (g/L)	37.031 ± 4.45	37.08 ± 4.28	0.061	0.805
HB (g/L)	128.96 ± 15.92	129.54 ± 16.83	1.250	0.264
PLT (10^9^/L)	210.51 ± 63.72	216.69 ± 66.49	1.251	0.264
WBC (10^9^/L)	8.35 (5.75, 10.41)	6.81 (4.87, 8.95)	−4.892	<0.001
NEU (10^9^/L)	6.23 (4.66, 8.46)	6.07 (4.43, 7.67)	−1.188	0.235
MO (10^9^/L)	0.77 (0.41, 1.36)	0.71 (0.36, 1.25)	−1.287	0.198
LY (10^9^/L)	2.14 (1.62, 2.64)	2.03 (1.54, 2.65)	−0.979	0.327
D-dimer (μg/mL)	1.42 (1.18, 1.67)	1.14 (1.02, 1.33)	−10.799	<0.001
FIB (g/L)	5.70 (3.37, 9.63)	4.99 (2.74, 7.58)	−3.848	<0.001
FDP (μg/mL)	14.40 (12.51, 16.15)	11.27 (9.55, 13.71)	−11.193	<0.001
ESR (mm/h)	42.98 ± 10.60	40.43 ± 8.90	10.332	0.001
CRP (mg/L)	56.55 ± 5.71	56.15 ± 5.60	0.007	0.936
Caprini (score)	12 (10, 14)	11 (8, 14)	−3.254	0.001
APTT (s)	29.64 (28.07, 32.39)	32.99 (31.28, 34.86)	−11.579	<0.001
PT (s)	13.14 ± 1.73	13.14 ± 1.78	0.062	0.0803

### 3.2 Comparison of data features between training set and verification set

The entire cohort was randomly divided into a training cohort (70%) and a validation cohort (30%). Baseline characteristics were compared between the training and validation cohorts to assess the comparability of the randomly divided datasets. Most variables were comparable between the two cohorts; however, diabetes, FDP, and APTT differed significantly between the training and validation cohorts. Although these differences may have introduced potential distributional heterogeneity, the nomogram retained good discriminative performance in the validation cohort, suggesting that the model had acceptable internal robustness. Nevertheless, this imbalance should be considered when interpreting the validation results, and external validation in independent cohorts is warranted (Table 3). Subsequently, the distribution of each factor within these groups and the corresponding analytical outcomes were evaluated for both the training and validation sets, as presented in Table 4.

**Table 3:** Comparison of baseline characteristics between the training and validation cohorts.

Variable	Training cohort (*n* = 635)	Validation cohort (*n* = 271)	*P* value
DVT *n* (%)	152 (23.94)	66 (24.35)	0.893
Gender *n* (%)	410 (64.57)	169 (62.36)	0.527
Affected side, right *n* (%)	341 (53.70)	133 (49.08)	0.202
Damage mechanism *n* (%)	313 (49.29)	146 (53.87)	0.206
Diabetes *n* (%)	339 (53.39)	125 (46.13)	0.045
Hypertension *n* (%)	339 (53.39)	139 (51.29)	0.563
Smoking history *n* (%)	344 (54.17)	136 (50.18)	0.271
Drinking history *n* (%)	327 (51.50)	151 (55.72)	0.244
Coronary disease *n* (%)	299 (47.09)	115 (42.44)	0.198
Operative approach *n* (%)	309 (48.66)	130 (47.97)	0.849
Anesthesia type *n* (%)	338 (53.23)	150 (55.35)	0.557
Transfusion *n* (%)	299 (47.09)	132 (48.71)	0.654
Age (years)	74.26 ± 7.25	73.60 ± 6.22	0.346
Injury-surgery time (days)	4.95 ± 2.06	4.99 ± 2.15	0.872
ALB (g/L)	37.05 ± 4.45	37.35 ± 4.01	0.326
HB (g/L)	129.40 ± 16.52	129.42 ± 16.85	0.984
PLT (10^9^/L)	215.04 ± 66.69	215.58 ± 63.98	0.911
WBC (10^9^/L)	7.30 ± 3.02	7.01 ± 3.20	0.126
NEU (10^9^/L)	6.24 ± 2.34	5.95 ± 2.63	0.161
MO (10^9^/L)	0.89 ± 0.66	0.86 ± 0.64	0.571
LY (10^9^/L)	2.09 ± 0.84	2.10 ± 0.80	0.917
D-dimer (µg/mL)	1.25 ± 0.24	1.37 ± 0.63	0.060
FIB (g/L)	5.73 ± 3.80	5.71 ± 3.62	0.907
FDP (µg/mL)	11.87 ± 3.42	13.30 ± 3.36	<0.001
ESR (mm/h)	41.14 ± 9.30	40.82 ± 9.62	0.641
CRP (mg/L)	56.21 ± 5.33	56.35 ± 6.28	0.740
Caprini (score)	11.12 ± 3.80	11.05 ± 3.78	0.809
BMI(kg/m^2^)	24.72 ± 2.28	24.92 ± 2.29	0.227
APTT (s)	32.17 ± 3.03	32.97 ± 3.34	<0.001
PT (s)	13.15 ± 1.75	13.12 ± 1.81	0.790

**Table 4:** Comparison of clinical characteristics between DVT and Non-DVT patients in the training and validation cohorts.

Variables	Train group			Test group		
	DVT (*n* = 152)	Non-DVT (*n* = 483)	*P*	DVT (*n* = 66)	Non-DVT (*n* = 205)	*P*
Age(years)	76 (70, 80)	73 (68, 79)	0.036	76 (71, 80)	72 (69, 77)	0.004
Gender *n* (%)			0.345			0.735
Female	49	176		26	76	
Male	103	307		40	129	
Affected side *n* (%)			0.529			0.863
Left side	67	227		33	105	
Right side	85	256		33	100	
Damage mechanism *n* (%)			0.587			0.900
High energy	72	241		36	110	
Low energy	80	242		31	95	
BMI(Kg/m^2^)	26.31 ± 1.95	24.22 ± 2.15	0.144			
Diabetes *n* (%)			<0.001			0.007
Yes	100	239		40	85	
No	52	244		26	120	
Hypertension *n* (%)			0.010			0.021
Yes	95	244		42	97	
No	57	239		24	108	
Smoking history *n* (%)			0.003			0.012
Yes	98	246		42	94	
No	54	237		24	111	
Drinking history *n* (%)			0.379			0.229
Yes	83	244		41	110	
No	69	239		25	95	
Coronary disease *n* (%)			0.632			0.773
Yes	69	230		27	88	
No	83	253		39	117	
Operative approach *n* (%)			0.705			0.219
THA	76	233		36	94	
HA	76	250		30	111	
Anesthesia type *n* (%)			0.839			0.203
CSEA	70	227		25	96	
General anesthesia	82	256		41	109	
Transfusion *n* (%)			0.052			0.543
Yes	82	217		30	102	
No	70	266		36	103	
Injury-surgery time (days)	5 (4, 6)	5 (3, 6)	0.874	5 (4, 7)	5 (3, 6.5)	0.307
ALB (g/L)	37.04 ± 4.42	37.9 ± 4.56	0.886	37.83 ± 4.15	37.19 ± 3.97	0.876
HB (g/L)	128.65 ± 15.36	129.63 ± 16.88	0.136	129.67 ± 17.26	129.34 ± 16.76	0.806
PLT (10^9^/L)	210.14 ± 63.12	216.59 ± 67.77	0.148	211.35 ± 65.57	216.94 ± 63.56	0.842
WBC (10^9^/L)	8.35 (6.01, 10.28)	6.91 (5.06, 9.20)	<0.001	8.26 (5.45, 10.72)	6.60 (4.62, 8.64)	0.003
NEU (10^9^/L)	6.38 (4.73, 8.28)	6.09 (4.67, 7.69)	0.295	5.98 (4.15, 8.67)	6.02 (4.08, 7.54)	0.548
MO (10^9^/L)	0.77 (0.41, 1.37)	0.73 (0.37, 1.26)	0.383	0.78 (0.33, 1.36)	0.70 (0.36, 1.24)	0.300
LY (10^9^/L)	2.19 (1.63, 2.67)	2.01 (1.54, 2.62)	0.179	2.09 ± 0.76	2.11 ± 0.81	0.651
D-dimer (μg/mL)	1.38 (1.16, 1.63)	1.14 (1.02, 1.31)	<0.001	1.47 (1.26, 1.85)	1.14 (1.02, 1.36)	<0.001
FIB (g/L)	5.60 (2.97, 9.82)	5.09 (2.73, 7.43)	0.007	6.92 (3.75, 9.21)	4.69 (2.71, 8.02)	0.006
FDP (μg/mL)	13.87 (12.15, 15.96)	11.01 (9.02, 13.50)	<0.001	15.01 (13.17, 16.42)	12.00 (10.18, 15.24)	<0.001
ESR (mm/h)	43.93 ± 10.58	40.27 ± 8.69	0.001	40.83 ± 10.42	40.82 ± 9.37	0.606
CRP (mg/L)	56.40 ± 5.34	56.14 ± 5.33	0.652	56.90 ± 6.52	56.17 ± 6.20	0.549
Caprini (score)	12 (9, 14)	11 (8, 14)	0.022	12 (10, 14)	11 (8, 13)	0.014
APTT (s)	29.49 (28.07, 31.60)	32.79 (31.20, 34.50)	<0.001	30.34 (27.77, 33.94)	33.67 (31.74, 35.75)	0.535
PT (s)	13.21 ± 1.59	13.13 ± 1.80	0.138	12.97 ± 2.01	13.17 ± 1.74	0.086

### 3.3 Variable filtering

**Figure 2:**
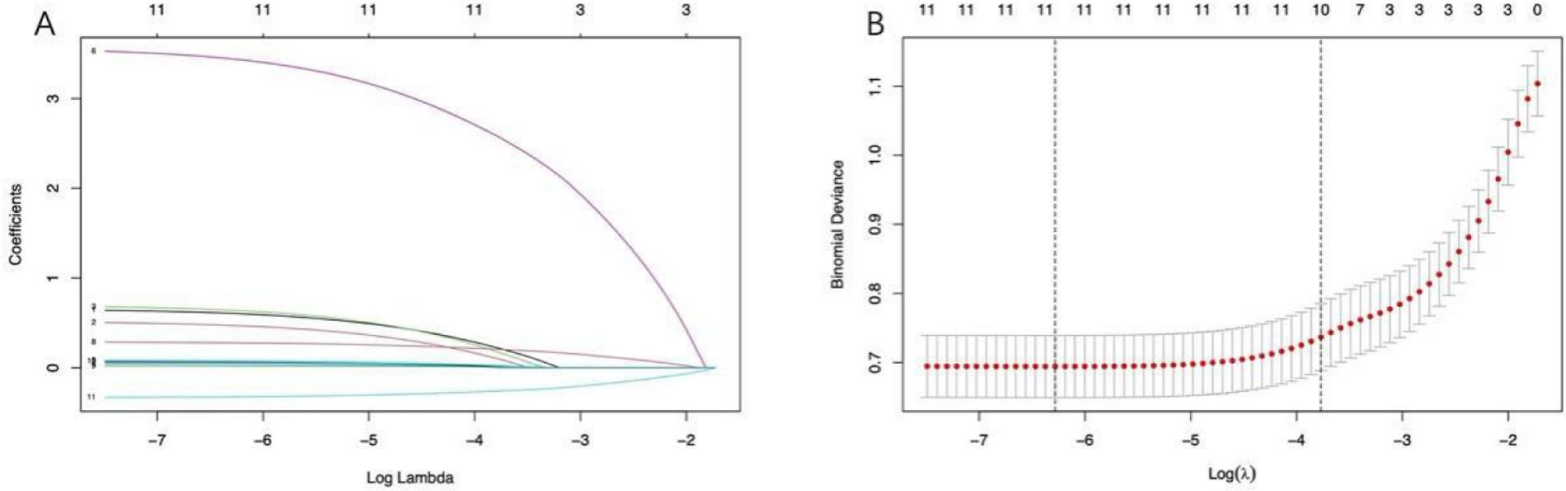
LASSO regression model used to screen the predictor variables. **(A)** LASSO coefficient profiles of the 10 features. A coefficient profile plot was produced against the log(l) sequence; **(B)** Relationship between the log(l) and the mean-squared error in the LASSO regression. Dotted vertical lines were drawn at the optimal values using the minimum criteria and the 1 Standard Error of the minimum criteria.

In the training dataset, the presence of DVT, coded as Yes = 1 and No = 0, served as the dependent variable. A LASSO regression analysis was subsequently conducted on the 11 independent variables that were statistically significant, as identified through univariate analysis. As the penalty coefficient (*λ*) was adjusted, the number of variables retained in the model progressively decreased. The optimal model was selected based on the minimum *λ* value plus one standard error (1SE = 0.023), as determined by the 10-fold cross-validation error. The final predictive variables incorporated into the model included diabetes, hypertension, smoking history, age, white blood cell count, D-dimer, FIB, FDP, erythrocyte sedimentation rate, and APTT) (refer to Figure 2). A multivariate logistic regression analysis was conducted on the selected predictors, revealing several significant factors associated with the development of DVT in patients with femoral neck fractures. The identified factors included diabetes (OR =  1.876, 95% CI = 1.144–3.078), hypertension (OR = 1.736, 95% CI = 1.058–2.848), smoking history (OR = 1.999, 95% CI = 1.217–3.284), age (OR = 1.040, 95% CI = 1.006–1.075), D-dimer levels (OR = 39.377, 95% CI = 14.133–109.713), FDP (OR = 1.350, 95% CI = 1.238–1.471), and APTT (OR = 0.710, 95% CI = 0.650–0.775). These findings underscore the primary risk factors for DVT in this patient cohort (refer to Table 5).

**Table 5:** Results of univariate logistic regression analysis of postoperative DVT complication in elderly patients with FNF.

Variables	*B*	Wald	OR	95% CI	*P*
Diabetes	0.629	6.207	1.876	1.144–3.078	0.013
Hypertension	0.552	4.772	1.736	1.058–2.848	0.029
Smoking	0.693	7.472	1.999	1.217–3.284	0.006
Age	0.039	5.244	1.040	1.006–1.075	0.022
D-dimer	3.673	49.363	39.377	14.133–109.713	0.000
FDP	0.300	46.547	1.350	1.238–1.471	0.000
APTT	−0.343	58.430	0.710	0.650–0.775	0.000

### 3.4 Construct a nomogram prediction model

**Figure 3:**
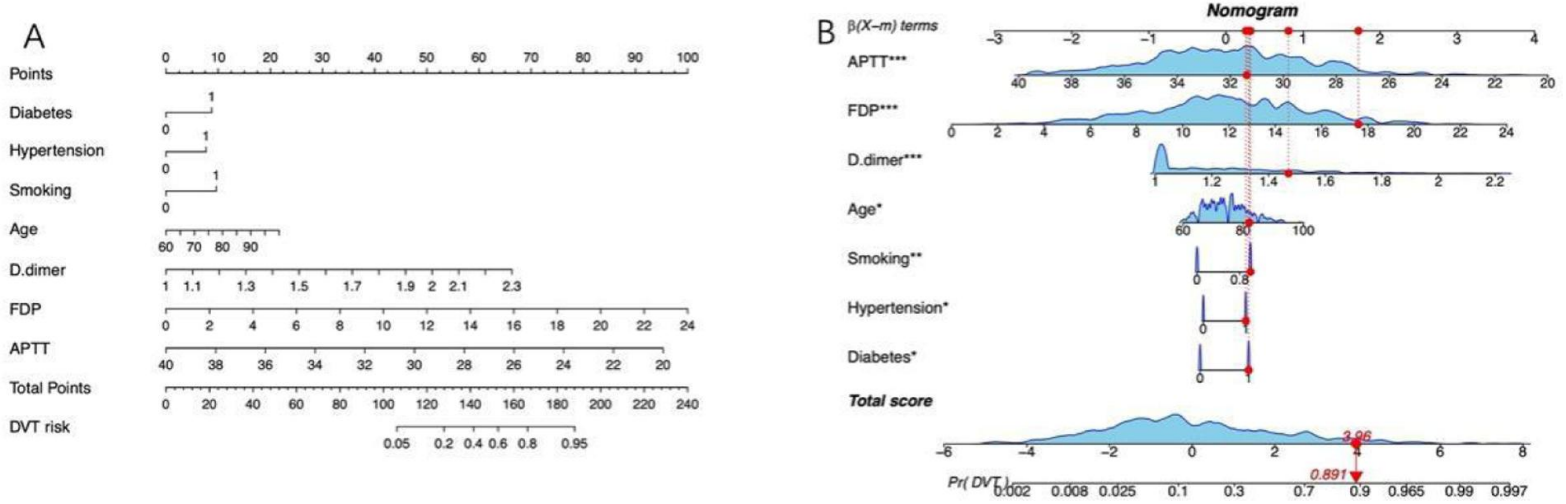
Nomogram for predicting the risk of DVT in patients with FNF. **(A)** Developed nomogram integrates significant predictors (age, diabetes, hypertension, smoking history, D-dimer, FDP, and APTT) to visually estimate DVT risk in individual patients. **(B)** Example illustrating the calculation of the total nomogram score and corresponding DVT probability for an individual patient case drawn from the cohort data.

The variables identified through multivariate logistic regression analysis were integrated into a nomogram prediction model. Subsequently, a nomogram was constructed to visually represent these predictors and their influence on the risk of developing DVT (Figure 3A). Each identified risk factor was assigned a score according to the scale provided in the nomogram. The total score was derived by summing the individual scores for all risk factors, which then indicated the probability of developing DVT. For illustrative purposes, a patient with a femoral neck fracture was randomly selected from the database. This patient exhibited the following risk factors: diabetes (score: 8.7), hypertension (score: 7.7), smoking history (score: 9.6), age 82 (score: 10.8), D-dimer level of 1.47 (score: 25.5), FDP level of 17.59 (score: 75), and APTT of 31.38 (score: 38.1). The cumulative score for this patient was approximately 175.4. Based on this total score, the predicted probability of developing DVT for this patient was approximately 89.1% (Figure 3B).

### 3.5 Verification model

**Figure 4:**
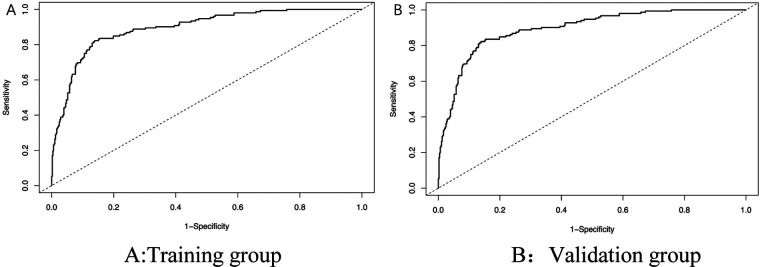
ROC curve of the nomogram prediction model to predict the risk of DVT. ROC analyses are displayed for both **(A)** the training cohort and **(B)** the validation cohort, demonstrating strong discriminative performance as indicated by the area under the ROC curves (AUC).

**Figure 5:**
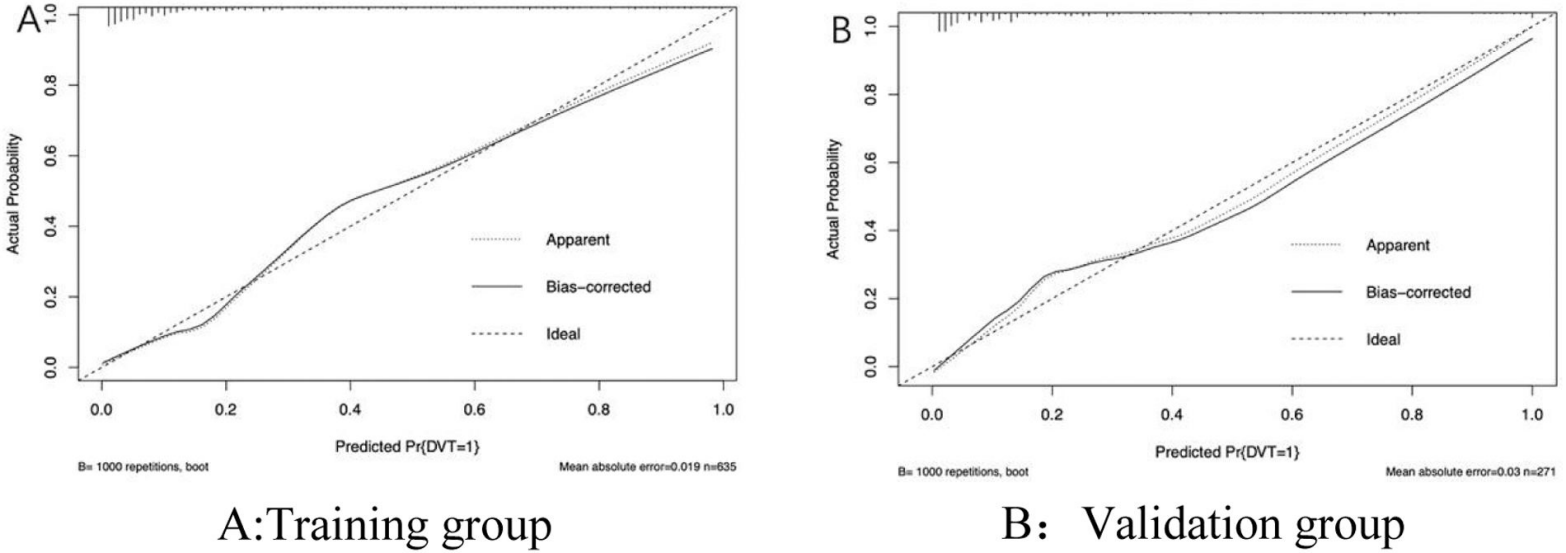
Hosmer–Lemeshow calibration curve of the prediction model. Hosmer–Lemeshow calibration plots are shown for the **(A)** training and **(B)** validation cohorts, reflecting excellent predictive concordance and model calibration.

**Figure 6:**
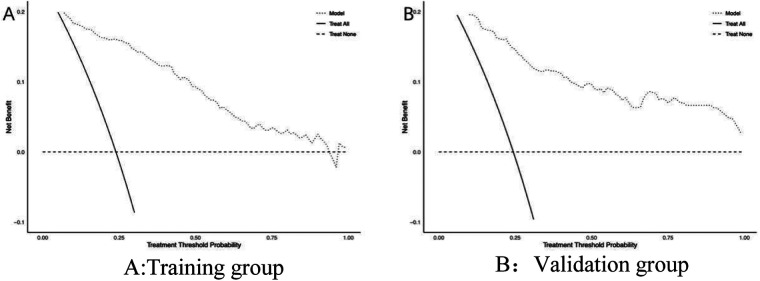
DCA analysis of the prediction model. Net benefit of applying the nomogram-based prediction model is displayed across a range of threshold probabilities for clinical decision-making in **(A)** training and **(B)** validation cohorts.

ROC curve analysis was performed to evaluate the discriminative ability of the nomogram for predicting the risk of DVT in patients with femoral neck fractures. In the training cohort, the model yielded an AUC of 0.894 (95% CI: 0.866–0.923; Figure 4A), indicating good discrimination. The optimal predicted probability cutoff, determined using the maximum Youden index, was 0.262, with a corresponding Youden index of 0.684. At this cutoff, the sensitivity, specificity, positive predictive value, and negative predictive value were 82.2%, 86.1%, 65.1%, and 93.9%, respectively. In the validation cohort, the model also demonstrated good discriminative performance, with an AUC of 0.900 (95% CI: 0.861–0.939; Figure 4B). Using the maximum Youden index in the validation cohort, the optimal cutoff was 0.156, corresponding to a Youden index of 0.597. At this cutoff, the sensitivity, specificity, positive predictive value, and negative predictive value were 90.9%, 68.8%, 48.4%, and 95.9%, respectively. In addition, when the training-derived cutoff of 0.262 was applied to the validation cohort, the model retained acceptable predictive performance, further supporting the robustness and potential generalizability of the nomogram. The Hosmer–Lemeshow goodness-of-fit test yielded non-significant results (*P* > 0.05), indicating that the model adequately fits the observed data. These findings were further supported by analyses conducted on the validation dataset (refer to Figure 5B). DCA was utilized to assess the clinical utility of the predictive model by calculating the net benefit across various threshold probabilities. The DCA results indicated that the model provided a positive net benefit to patients within a threshold probability range of 0–0.95 (see Figure 6A), a finding that was corroborated in the validation cohort (see Figure 6B). The extensive range of acceptable threshold probabilities underscores the model's utility as a valuable tool for assessing the risk of DVT.

## 4 Discussion

In the present study, we developed and internally validated a nomogram for predicting postoperative DVT in patients with femoral neck fractures undergoing hip arthroplasty. Based on LASSO regression and multivariate logistic regression analyses, seven independent predictors were ultimately identified, including age, diabetes, hypertension, smoking history, D-dimer, FDP, and APTT. The model demonstrated good discriminative ability in both the training cohort (AUC = 0.894, 95% CI: 0.866–0.923) and validation cohort (AUC = 0.900, 95% CI: 0.861–0.939), indicating favorable predictive performance. Furthermore, calibration and decision curve analyses confirmed satisfactory agreement between predicted and observed outcomes and suggested potential clinical utility.

Age was identified as an independent predictor of postoperative DVT in the present study (OR = 1.040, 95% CI: 1.006–1.075). This finding suggests that the risk of DVT increased by approximately 4% with each additional year of age. Considering that the mean age of our cohort exceeded 74 years, age-related endothelial dysfunction, reduced venous return secondary to prolonged immobilization, and impaired fibrinolytic activity may collectively contribute to thrombus formation. Similar associations between advanced age and DVT have been reported in patients with hip fractures and other orthopedic conditions (5). Age has also been identified as an independent predictor of DVT in both retrospective and prospective studies involving elderly fracture patients (18, 19). Therefore, age should be regarded as an important component of perioperative thrombosis risk assessment in patients with femoral neck fractures.

Diabetes was another significant predictor retained in the final model (OR = 1.876, 95% CI: 1.144–3.078), indicating an approximately 88% increase in postoperative DVT risk among diabetic patients. In elderly patients with femoral neck fractures, chronic hyperglycemia may exacerbate endothelial injury, platelet activation, inflammatory responses, and abnormalities in fibrinolysis, thereby promoting a hypercoagulable state (20). Our findings are consistent with previous studies demonstrating that diabetes is independently associated with postoperative DVT following hip fracture surgery (21). Similar results have been reported in prospective cohort studies and meta-analyses, which showed that diabetes significantly increases the risk of postoperative venous thromboembolism (19, 22). These findings suggest that perioperative glycemic control may represent an important strategy for reducing thrombotic complications in this population.

Hypertension was independently associated with DVT occurrence in our cohort (OR = 1.736, 95% CI: 1.058–2.848). Long-standing hypertension may impair vascular endothelial integrity and promote coagulation activation, thereby facilitating thrombus formation (23). In elderly patients with femoral neck fractures, the coexistence of hypertension, vascular aging, and perioperative immobilization may further amplify this risk. Our results are consistent with a meta-analysis demonstrating that hypertension is associated with a significantly increased risk of DVT after orthopedic surgery (24). Although Mendelian randomization studies have reported inconsistent findings regarding the causal relationship between hypertension and venous thrombosis (25), the present study supports the clinical relevance of hypertension as a risk factor in this specific patient population.

Smoking history was associated with an almost twofold increase in postoperative DVT risk (OR = 1.999, 95% CI: 1.217–3.284). Cigarette smoking can enhance platelet aggregation, increase oxidative stress, and impair endothelial function, thereby promoting a prothrombotic environment (26). In our cohort, smoking remained significant after adjustment for multiple confounding variables, suggesting that its effect was independent of other clinical factors. Previous epidemiological studies and meta-analyses have similarly reported a positive association between smoking and venous thromboembolism (27, 28). However, conflicting findings have been reported by Mendelian randomization analyses and some observational studies (29, 30). The discrepancy may reflect differences in study populations, smoking exposure assessment, and underlying comorbidities. Nevertheless, given that smoking is a modifiable risk factor, smoking cessation should be encouraged during perioperative management.

Among all predictors included in the nomogram, D-dimer demonstrated the strongest association with postoperative DVT (OR = 39.377, 95% CI: 14.133–109.713). This remarkably high odds ratio suggests that activation of coagulation and fibrinolytic pathways plays a central role in thrombus formation following femoral neck fractures. Tissue trauma, surgical stress, inflammatory responses, and prolonged immobilization may substantially increase fibrin formation and degradation, resulting in elevated D-dimer concentrations. Previous studies have consistently shown that elevated D-dimer levels are strongly associated with DVT development and may serve as an important marker of thrombotic activity (31, 32). Furthermore, elevated D-dimer levels have been shown to predict venous thromboembolism in hospitalized orthopedic patients (33). The present findings further support the clinical value of D-dimer as one of the most powerful biomarkers for identifying patients at high risk of postoperative DVT.

FDP was also independently associated with DVT occurrence (OR = 1.350, 95% CI: 1.238–1.471). As a degradation product generated during fibrinolysis, FDP reflects ongoing thrombus formation and fibrin degradation. Notably, both FDP and D-dimer remained significant in the final model, suggesting that dynamic changes in coagulation and fibrinolytic activity provide important prognostic information beyond conventional clinical characteristics. Previous studies have demonstrated elevated FDP concentrations in patients with DVT and have reported significant correlations between FDP and D-dimer levels in hypercoagulable states (34, 35). In addition, FDP has been identified as an independent predictor of DVT in orthopedic patients (36). These findings support the combined use of FDP and D-dimer in perioperative thrombotic risk assessment.

APTT was the only variable inversely associated with DVT risk (OR = 0.710, 95% CI: 0.650–0.775), indicating that shorter clotting times were associated with a higher probability of thrombosis. In our study, patients who developed DVT exhibited elevated D-dimer and FDP levels together with reduced APTT values, collectively suggesting an activated coagulation profile. Shortened APTT may reflect enhanced activation of the intrinsic coagulation pathway and a hypercoagulable state, thereby predisposing patients to thrombus formation. Similar findings have been reported in previous studies, in which reduced APTT was identified as an independent predictor of DVT (37). Therefore, routine coagulation parameters may provide valuable information for early thrombotic risk stratification in elderly patients with femoral neck fractures.

Beyond identifying independent risk factors, the major strength of the present study lies in the development of a clinically applicable nomogram integrating demographic characteristics, comorbidities, and coagulation-related biomarkers. The model demonstrated excellent discrimination in both the training and validation cohorts, with AUC values approaching 0.90, indicating a strong ability to distinguish between patients with and without DVT. Moreover, calibration curves demonstrated good agreement between predicted and observed outcomes, while decision curve analysis showed a positive net clinical benefit across a broad range of threshold probabilities. These findings suggest that the nomogram may facilitate individualized risk assessment and help clinicians identify patients who may benefit from intensified thromboprophylaxis and closer postoperative monitoring.

Limitations of this study are as follows: Firstly, it was conducted as a single-center, retrospective case-control study with a relatively small sample size, which may introduce confounding factors and constrain the robustness of the evidence. Secondly, the model underwent only internal validation; thus, external validation using additional datasets is essential to confirm and enhance its reliability. Thirdly, the study employed multiple exclusion criteria, such as pathological fractures, thrombotic diseases, use of anticoagulants, certain DVT-related conditions, and delayed hospital admission, which further restricted the sample size. Lastly, data collection was limited to 29 influencing factors from patients within our hospital, potentially omitting other factors that could have impacted the results. Future research involving larger sample sizes and extended follow-up periods is warranted to further investigate the factors influencing the occurrence of DVT.

To address the above limitation, we plan to conduct multicenter external validation studies. We will collaborate with other institutions to prospectively collect data from diverse patient populations. The established nomogram will be tested on independent external cohorts to evaluate its discriminative ability (AUC), calibration, and clinical utility.

## 5 Conclusion

Utilizing patient clinical data and laboratory results, we have successfully developed and validated a novel, precise, and efficacious nomogram for predicting the incidence of DVT following FNF, employing straightforward influencing factors. This model facilitates the assessment of DVT risk by analyzing these factors, thereby enabling physicians to more accurately identify patients at high risk and implement timely and effective preventive interventions. The development and validation of this model represent a significant advancement in reducing the incidence of DVT post-FNF and enhancing patient outcomes. Future research endeavors will focus on further optimizing this model, thereby augmenting its utility as a valuable tool for clinicians to deliver safer and more personalized patient care.

## Data Availability

The raw data supporting the conclusions of this article will be made available by the authors, without undue reservation.
